# Case report: Novel transtemporal transverse sinus decompression surgery to alleviate transverse sinus stenosis in a pulsatile tinnitus patient with restricted bilateral venous outflow

**DOI:** 10.3389/fsurg.2023.1268829

**Published:** 2023-09-28

**Authors:** Yue-Lin Hsieh, Wuqing Wang

**Affiliations:** ENT Institute and Department of Otorhinolaryngology, Eye & ENT Hospital, Fudan University, Shanghai, China

**Keywords:** pulsatile tinnitus, extraluminal surgery, transverse sinus stenosis (TSS), sinus decompression, increased intracranial pressure (ICP), idiopathic intracranial hypertension (IIH)

## Abstract

Transverse sinus (TS) stenosis is common in individuals with venous pulsatile tinnitus (PT). While PT can be addressed by endoluminal or extraluminal methods, the former has shown promise in alleviating symptoms associated with increased intracranial pressure. This study explores the potential of extraluminal methods to alleviate TS stenosis and eliminate PT caused by sigmoid sinus diverticulum. A 31-year-old male patient presenting with left-sided PT, attributed to a large, pedunculated sigmoid sinus diverticulum along with severe ipsilateral TS stenosis and contralateral TS hypoplasia, underwent ipsilateral extraluminal TS decompression surgery following sigmoid sinus wall reconstruction under local anesthesia. Postoperative CT and MR angiography revealed a significant increase in the TS lumen from 0.269 to 0.42 cm^2^ (56.02%) 2 years after surgery. Cervical Doppler ultrasound demonstrated a 36.07% increase in ipsilateral outflow volume to 16.6 g/s and a 77.63% increase in contralateral outflow volume to 1.35 g/s. In conclusion, this pioneering study showcases the potential of transtemporal TS decompression surgery in creating space for adaptive expansion of the TS lumen. However, the procedure should be reserved for individuals with severely compromised venous return.

## Introduction

Vascular pulsatile tinnitus (PT) is characterized by an unusual perception of blood flow sounds synchronized with the heartbeat (1). Among the various types of vascular PT, venous PT is the most common and is often linked to sigmoid sinus wall anomalies (SSWAs) and transverse sinus (TS) stenosis, which are frequently targeted by surgical interventions to alleviate PT (2, 3).

SSWAs include sigmoid sinus diverticulum (SSD) and wall dehiscence, both of which are believed to result from distal TS stenosis and elevated intracranial pressure commonly observed in individuals with idiopathic intracranial hypertension (IIH) (4). However, while TS stenosis is a common finding in the normal population, SSWAs are comparatively rare, making them more likely to be associated with PT generation (5).

Endoluminal stenting, primarily focusing on TS stenosis, has emerged as an effective treatment for PT and associated IIH symptoms (3, 6). Alleviating TS stenosis lowers regional blood flow and pressure gradients, resulting in decreased kinetic energy of blood flow and subsequent relief of PT (7). On the other hand, transtemporal sigmoid sinus wall reconstruction surgery aims to correct the defective bone dehiscence and minimize SSD, both of which have shown positive outcomes in PT surgery (2).

In this study, we present a case of PT featuring substantial SSD, severe ipsilateral distal TS stenosis, and contralateral TS aplasia. The surgical approach involved a combination of transtemporal sigmoid sinus wall reconstruction surgery and a novel TS decompression procedure to address PT and prevent inadequate intracranial venous return associated with SSD and distal TS stenosis.

## Case presentation

**Figure 1:**
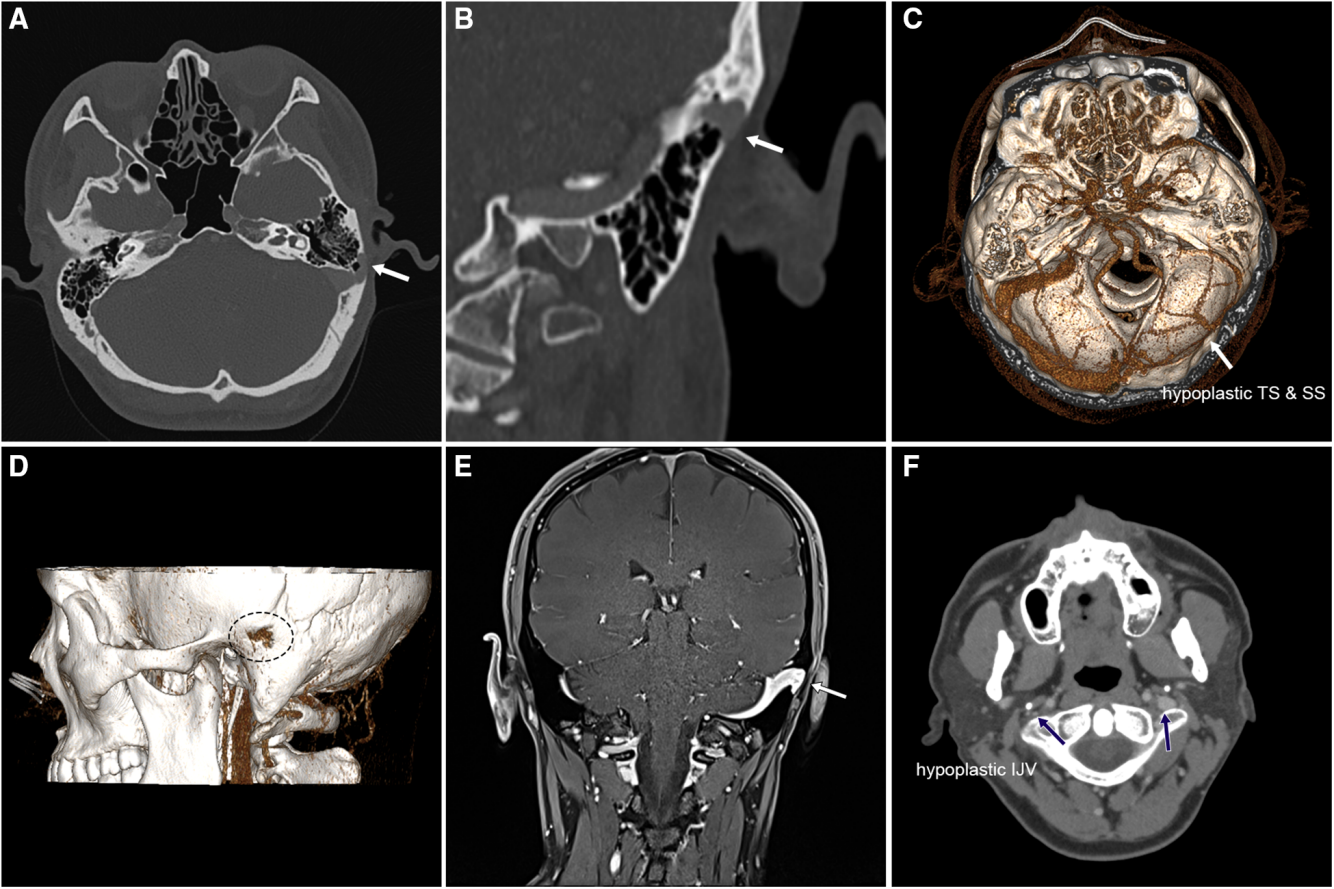
Preoperative contrast-enhanced CT and MR images of the case exhibiting a large, left-sided pedunculated protrusive diverticulum and ipsilateral distal TS stenosis coupled with contralateral TS hypoplasia. (**A**) Axial contrast-enhanced CT image demonstrating the protrusion of the pedunculated diverticulum into the subcutaneous tissue (white arrow). (**B**) Coronal contrast-enhanced CT showing the protrusion of the pedunculated diverticulum into the subcutaneous tissue (white arrow). (**C**) Volume rendering technique (VRT) of the CT angiography showcasing ipsilateral dominant drainage and contralateral TS hypoplasia. (**D**) Volume rendering technique of the CT angiography illustrating the location of erosion of the mastoid cortex and diverticular wall protrusion caused by the diverticulum. (**E**) Coronal contrast-enhanced MR image exhibiting the pedunculated diverticulum and underdeveloped contralateral sigmoid sinus. (**F**) Axial contrast-enhanced CT slice revealing a significantly smaller internal jugular vein on the left side than on the right side (blue arrows).

**Figure 2:**
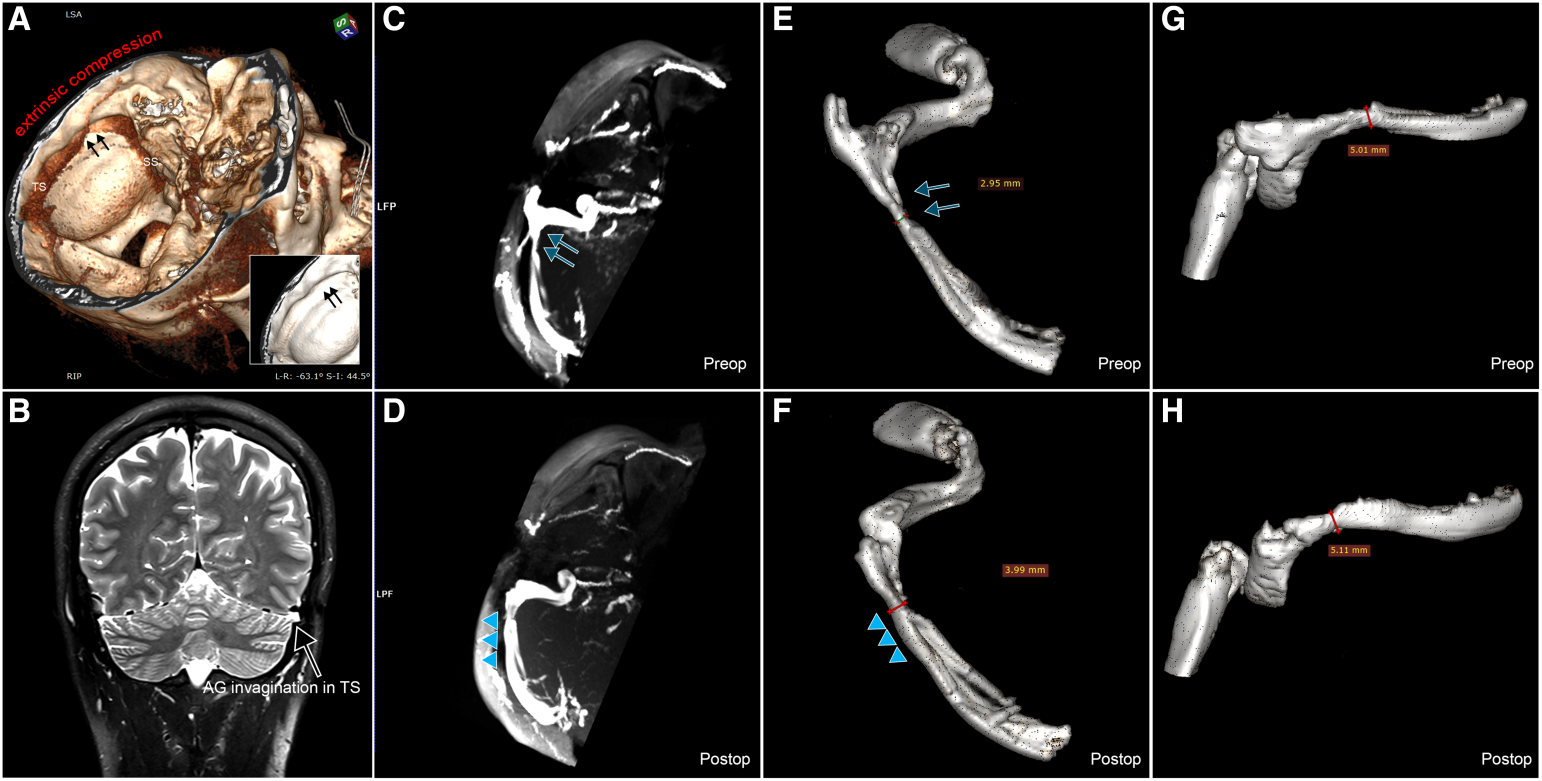
Preoperative and postoperative CT and MR images demonstrating TS lumen expansion. (**A**) Preoperative VRT CT image revealing extrinsic compression of the brain parenchyma (indicated by black arrows). (**A**) T2 coronal MR image illustrating intrinsic compression of the TS lumen due to arachnoid granulation (AG) invagination. (**C**) Preoperative maximum intensity projection (MIP) MR venogram image showcasing the TS stenosis in the case (highlighted by blue arrows). (**D**) Postoperative MIP MR venogram image displaying the enlargement of the TS lumen and the relief of the TS stenosis (indicated by blue triangles). (**E**) Preoperative VRT image reconstructed using the MR venogram of the ipsilesional transverse-sigmoid sinus without communicating branches (blue arrows). (**F**) Postoperative VRT image reconstructed using the MR venogram of the ipsilesional transverse-sigmoid sinus without communicating branches (blue triangles). A significant lateral expansion of the postoperative TS lumen can be observed compared to the preoperative condition. (**G**) Preoperative VRT image reconstructed using the MR venogram of the ipsilesional transverse-sigmoid sinus. (**H**) Postoperative VRT image reconstructed using the MR venogram of the ipsilesional transverse-sigmoid sinus. Here, a slight difference in the vertical expansion of the postoperative TS lumen can be discerned compared to the preoperative state.

A 31-year-old male patient presented to our PT clinic with a complaint of left-sided PT persisting for 4 years. Upon further consultation, mild intermittent headaches were reported. Compression of the internal jugular vein (IJV) resulted in immediate subsidence of PT, and a positive water occlusion test indicated a venous etiology associated with SSWAs. Contrast-enhanced computed tomography (CT) and magnetic resonance (MR) imaging were performed, revealing a large left-sided SSD, severe ipsilateral distal TS stenosis, and contralateral transverse-sigmoid sinus aplasia (Figures 1, 2). Upper IJV demonstrated outflow volumes of 12.2 and 0.76 g/s on the ipsilateral and contralateral sides, respectively. Fundoscopy and orbital ultrasound examinations ruled out signs of papilledema. The open lumbar pressure was 250 mmH_2_O. According to prior research, an intrinsic TSS was defined as a localized occlusion within the TS lumen and was primarily attributed to the presence of arachnoid granulations (8), whereas an extrinsic TSS was defined as a gradual constriction affecting a relatively extensive segment. The transtemporal TS decompression surgery was aimed at treating the TSS in our case, which was brought on by both intrinsic arachnoid granulation invagination and extrinsic brain parenchymal compression.

**Figure 3:**
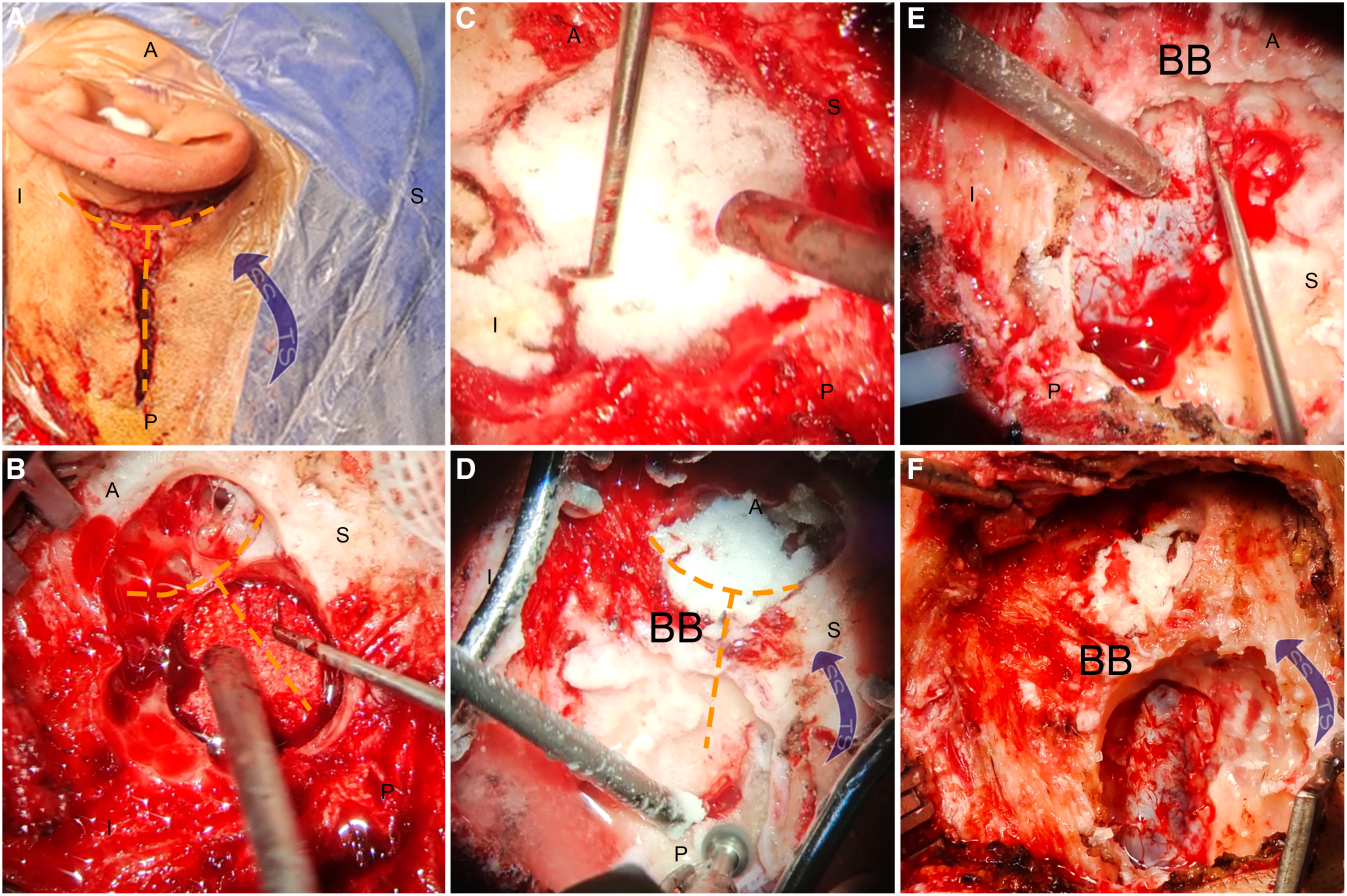
Preoperative and postoperative (2 years) radiologic outcomes of transtemporal TS decompression surgery. (**A,B**) Corresponding coronal contrast-enhanced CT images showcasing the most significant postoperative expansion of the cross-sectional area of the TS at the distal end of the TS. (**C,D**) Expansion of the TS lumen at the site of intrinsic and extrinsic compression (indicated by white arrows) following surgical decompression (indicated by yellow arrows). It is noteworthy that the presence of the BB prevents the subcutaneous tissue from collapsing the TS wall. (**E,F**) Postoperative expansion of the TS lumen after surgical decompression (yellow arrows).

To perform sigmoid sinus wall reconstruction and transtemporal TS decompression surgery (Figure 3), a Y-shaped incision is required that intersects with the postauricular C-shaped incision. The transtemporal TS decompression surgery follows sigmoid sinus wall reconstruction. The method of sigmoid sinus wall reconstruction surgery is identical to our previous techniques (9). TS is visualized and exposed by extending the skeletonization of the SSD superiorly. The precise location of TS stenosis can be determined by measuring the distance between the SSD and the stenotic region to facilitate the complete expansion of the TS lumen. The skeletonization of the TS and separation of the vascular wall should extend up to 0.5 mm beyond the narrowest section of the stenosis. Furthermore, the overlying bone structure of the TS surface is typically removed to minimize resistance to expansion. It is important to mention that a bridging bone (BB) is preserved for support, and the lateral bone surface is left unreconstructed to prevent the collapse of the delicate TS wall by the applied materials. Moreover, it is crucial to proactively identify diploic and/or emissary veins to mitigate the risk of intraoperative hemorrhage and ensure the safety of the TS decompression surgery.

## Results

**Figure 4:**
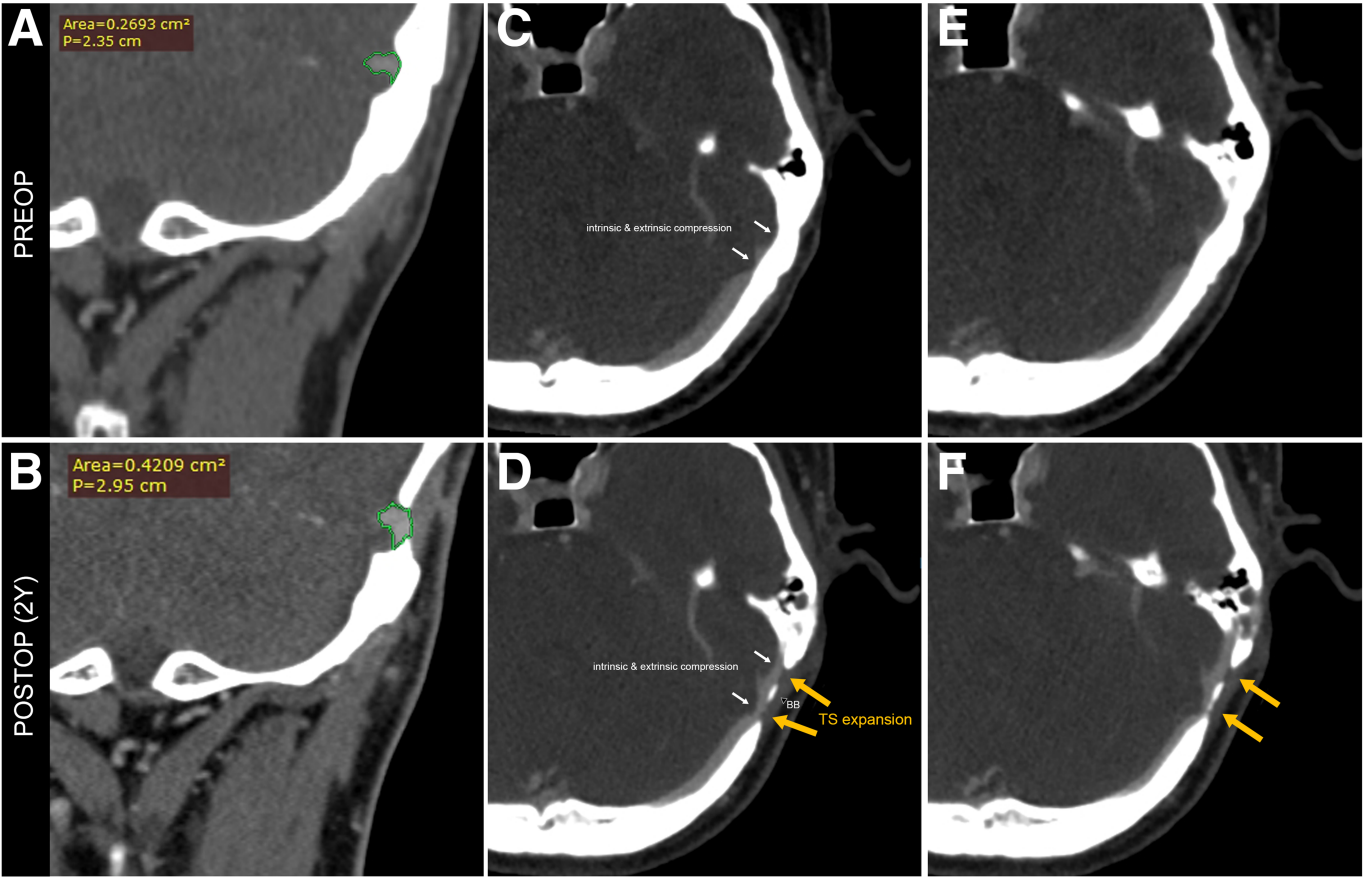
Intraoperative photograph showing the transtemporal TS decompression surgery following sigmoid sinus wall reconstruction surgery under local anesthesia. (**A**) A “Y”-shaped incision was made to initiate the transtemporal TS decompression surgery alongside the sigmoid sinus wall reconstruction surgery. (**B**) The sigmoid sinus diverticulum was reduced and resurfaced using a solidified gel foam brick. (**C**) Further enhancement of the sigmoid sinus wall was performed using solidified gel foam. (**D**) Skeletonization of the TS was carried out while preserving a BB to prevent the collapse of the TS wall caused by reapproximated subcutaneous tissue. (**E**) The dura and vascular wall were separated from the surrounding osseous walls to decompress the TS lumen. (**F**) The final outcome of the transtemporal TS decompression surgery coupled with sigmoid sinus wall reconstruction surgery. A, anterior; P, posterior; S, superior; I, inferior; BB, bridging bone; SS, sigmoid sinus; TS, transverse sinus.

PT resolved immediately and completely after the sigmoid sinus wall reconstruction procedure under local anesthesia prior to the TS decompression surgery. Throughout the comprehensive 2-year follow-up period, no recurrence of PT was observed. The patient's intermittent headaches also resolved during the 7-month postoperative follow-up, as evidenced by the numeric rating scale, where scores ranging from 4 to 6 (indicating moderate pain) were reduced to a score of 0 (signifying the absence of a headache). The TS lumen at the most stenotic segment exhibited a significant increase, growing from 0.269 to 0.42 cm^2^ (a 56.02% increase) 2 years after surgery (Figure 4). Ipsilateral outflow volume increased to 16.6 g/s (36.07%) and contralateral outflow volume increased to 1.35 g/s (77.63%). No postoperative complications were reported, and there was no postsurgical medication was prescribed to the subject. The postoperative observation period was extended for 2 days, and then the patient was subsequently discharged.

## Discussion

The development of transtemporal TS decompression surgery was primarily motivated by the presence of SSD accompanied by severe ipsilateral distal TS stenosis and contralateral transverse-sigmoid sinus aplasia. The surgical procedure aimed to reduce intracranial pressure by decreasing the size of the diverticulum and to improve venous drainage by removing the lateral temporal bone structure for lateral expansion of the TS wall.

The non-invasive confirmation of TS stenosis necessitates the utilization of either CT or MR angiography. CT angiography has the advantage of simultaneously delineating temporal bone and vascular structures while avoiding filling defects. Conversely, MR has demonstrated notable efficacy and non-invasiveness in detecting both intrinsic and extrinsic TS stenosis, along with higher sensitivity in revealing dural arteriovenous fistulas (10, 11). It is noteworthy that while 4D-flow MR may be considered preferable for exploring surgical velocity variations, its application demands a higher level of technical proficiency and is not incorporated in the present study. In contrast, digital substrate angiography and the implementation of pressure wire provide the most precise anatomical and pressure gradient information about TS stenosis (3); however, it is imperative to acknowledge that these procedures entail invasiveness, leading some of our patients to decline their utilization. On the other hand, Doppler ultrasound, having excellent time and spatial resolution (12), assumes a pivotal, non-invasive role as an adjunctive tool for the assessment of venous outflow volume and the augmentation of our comprehension of volumetric alterations in patients undergoing transtemporal TS decompression surgery. Hence, for a comprehensive exploration of anatomical variations in TS stenosis, it is advisable to employ contrast-enhanced CT and MR angiography.

Based on a CT angiogram, transtemporal TS decompression surgery resulted in a remarkable 56.02% increase in the cross-sectional area of the distal TS lumen. Furthermore, during a 7-month follow-up period, an improvement in headache symptoms was observed, likely attributed to the expansion of the TS and subsequent alleviation of the headache. These radiologic and postoperative changes highlight the efficacy, adaptability, and, importantly, safety of transtemporal TS decompression surgery as an extraluminal surgical approach to address TS stenosis.

Regarding the categorization of TS stenosis types, some scholarly perspectives have advocated for the need to distinguish between intrinsic and extrinsic stenosis (8). Extrinsic stenosis may exert a more pronounced influence on venous outflow and intracranial pressure when compared to intrinsic stenosis in isolation (8). Despite our omission of intracranial pressure monitoring post-TS decompression surgery and the absence of pressure gradient assessment throughout our study pursuing minimal invasiveness, it is theoretically conceivable that facilitating lateral expansion of the stenotic TS lumen could enhance flow patency. This potential improvement is irrespective of the specific stenosis type since intrinsic and extrinsic TS stenosis variants medially compress the TS lumen. Consequently, improvement of headache symptoms, enlarged TS lumen, and ipsilateral intracranial outflow volume measured by Doppler ultrasound may indicate enhanced sinus hemodynamics subsequent to TS decompression surgery.

Compared to the endoluminal method, the TS decompression technique does not involve manipulation of TS hemodynamics within the lumen. As a result, postoperative medication is not required, and the risk of in-stent thrombosis, laceration of the transverse sinus septum, and stent-adjacent stenosis is minimized. However, it should be noted that the duration of sinus wall expansion may impact postoperative headache symptom relief and may also reduce regional velocity due to the Bernoulli effect. However, since most Asian individuals with isolated PT associated with SSWAs do not meet the criteria for a diagnosis of IIH (13), it is advisable to reserve this procedure for individuals with SSWA-associated PT who have suspected or confirmed IIH or those with severely compromised transverse-sigmoid sinus outflow.

While this study is rooted in a case report, we undertook a 2-year postoperative monitoring period to assess and validate the expansion of the TS lumen. Nevertheless, it is important to acknowledge that the assessment of hemodynamic improvement could potentially be further enhanced by incorporating additional methodologies. These may include postoperative cerebrospinal fluid manometry or intraluminal measurement of trans-stenotic pressure gradients. However, it should be emphasized that these supplementary examinations are invasive in nature and, as such, were not included in the scope of this particular study. Furthermore, this study introduces prospects for tackling distal TS stenosis associated with SSWAs through extraluminal methodologies, thereby opening avenues for the exploration and refinement of TS decompression/reconstruction techniques within a broader spectrum of related conditions.

## Conclusion

Beyond its role in the treatment of PT by sigmoid sinus wall reconstruction, transtemporal TS decompression surgery exhibits promise in facilitating the sustained expansion of the TS lumen, thus mitigating distal TS stenosis arising from both intrinsic and extrinsic compressions. Consequently, TS decompression surgery may be regarded as a supplementary surgical intervention when performed in conjunction with sigmoid sinus wall reconstruction surgery, to further ameliorate significantly impaired sinus hemodynamics alongside the primary objective of PT management. Nonetheless, it is advisable to limit this procedure to patients with severely compromised bilateral venous return. Further exploration of the broader implications and applications of this surgical approach holds promise for future advancements in the field.

## Data Availability

The original contributions presented in the study are included in the article/Supplementary Material, further inquiries can be directed to the corresponding author.
